# Altered Resting-State Electroencephalography Microstates in Idiopathic Generalized Epilepsy: A Prospective Case-Control Study

**DOI:** 10.3389/fneur.2021.710952

**Published:** 2021-11-22

**Authors:** YuBao Jiang, MingYu Zhu, Ying Hu, Kai Wang

**Affiliations:** ^1^Department of Neurology, The First Affiliated Hospital of Anhui Medical University, Hefei, China; ^2^The School of Mental Health and Psychological Sciences, Anhui Medical University, Hefei, China; ^3^Anhui Province Key Laboratory of Cognition and Neuropsychiatric Disorders, Hefei, China; ^4^Collaborative Innovation Center of Neuropsychiatric Disorders and Mental Health, Hefei, China; ^5^Institute of Artificial Intelligence, Hefei Comprehensive National Science Center, Hefei, China

**Keywords:** electroencephalography, microstates, generalized epilepsy, seizures, salience network, sensorimotor network

## Abstract

**Objective:** Idiopathic generalized epilepsy (IGE) involves aberrant organization and functioning of large-scale brain networks. This study aims to investigate whether the resting-state EEG microstate analysis could provide novel insights into the abnormal temporal and spatial properties of intrinsic brain activities in patients with IGE.

**Methods:** Three groups of participants were chosen for this study (namely IGE-Seizure, IGE-Seizure Free, and Healthy Controls). EEG microstate analysis on the resting-state EEG datasets was conducted for all participants. The average duration (“Duration”), the average number of microstates per second (“Frequency”), as well as the percentage of total analysis time occupied in that state (“Coverage”) of the EEG microstate were compared among the three groups.

**Results:** For microstate classes B and D, the differences in Duration, Frequency, and Coverage among the three groups were not statistically significant. Both Frequency and Coverage of microstate class A were statistically significantly larger in the IGE-Seizure group than in the other two groups. The Duration and Coverage of microstate class C were statistically significantly smaller in the IGE-Seizure group than those in the other two groups.

**Conclusions:** The Microstate class A was regarded as a sensorimotor network and Microstate class C was mainly related to the salience network, this study indicated an altered sensorimotor and salience network in patients with IGE, especially in those who had experienced seizures in the past 2 years, while the visual and attention networks seemed to be intact.

**Significance:** The temporal dynamics of resting-state networks were studied through EEG microstate analysis in patients with IGE, which is expected to generate indices that could be utilized in clinical researches of epilepsy.

## Highlights

- Resting-state EEG microstate analysis was used to determine the sub-second temporal brain activity in IGE patients.
- IGE patients showed altered sensorimotor and salience network, especially in those with a recent history of seizures.
- The visual and attention network were seemingly intact in IGE patients.

## Introduction

Epilepsy is one of the most common diseases of the central nervous system, characterized by recurrent and spontaneous seizures which result from excessive abnormal synchronous discharge of neurons. Idiopathic (or genetic) generalized epilepsy (IGE) is a well-recognized subgroup of generalized epilepsy, referring to four epilepsy syndromes: childhood absence epilepsy, juvenile absence epilepsy, juvenile myoclonic epilepsy, and generalized tonic–clonic seizures (GTCS) alone (1). GTCS is the most common type of seizures in IGE (2). Previously functional magnetic resonance imaging (fMRI) study indicated abnormal functional connectivity (FC) in default mode network (DMN), dorsal attention network (DAN), sensorimotor network (SMN), visual network (VN) and auditory network (AN), suggesting a large-scale brain network abnormity in patients with IGE (3–5). Recently, the temporal variability of FC has attracted increasing attention. Patients with GTCS demonstrated the reliable abnormality of FC temporal variability in cognition related functional networks, especially in the DMN, and increased FC temporal variability in motor-related regions (6). The dynamic FC analysis may help us to understand the pathophysiology mechanisms of GTCS.

**Figure 1:**
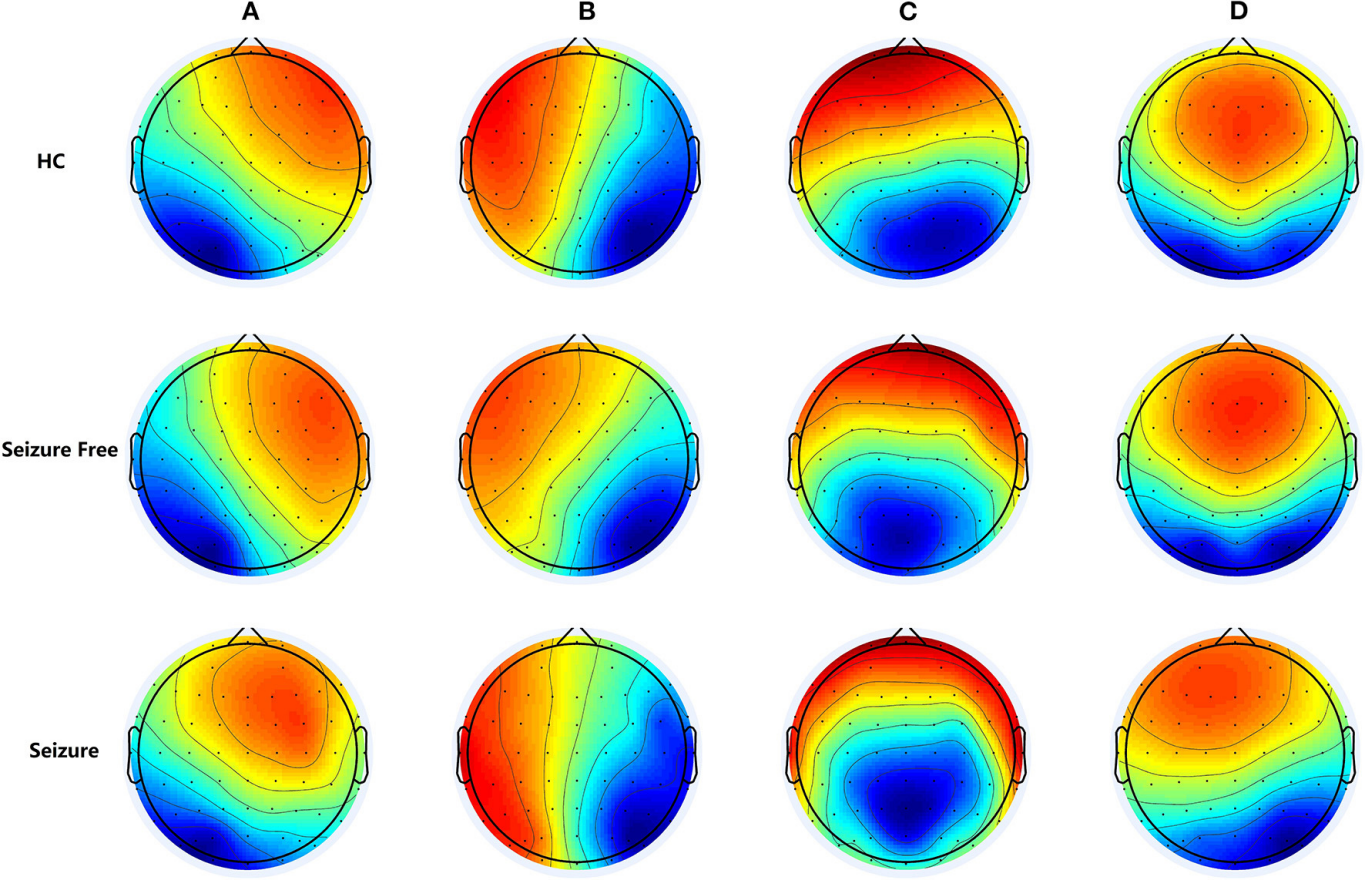
The group-level maps of the four microstate classes (A–D) of three groups. Topographical microstate maps of three groups: Microstate class A exhibits a left-right orientation, class B a right-left orientation, class C an anterior-posterior orientation, and class D a fronto-central maximum, these four cluster maps exhibited highly similar topographies in most previously studies.

The multichannel electroencephalogram (EEG) is a widely used technique with major clinical importance in the diagnosis of epilepsy. The postsynaptic inhibitory and excitatory potentials are the main sources of EEG signals. EEG is used to measure the synchronous electrical activity of spatially aligned neurons directly and dynamically in cortical networks. Therefore, EEG captures useful information on brain activity. Topographical map of electrical potentials characterizes the EEG signals by the spatial configuration of the electric fields at the scalp, which considers the information of all electrodes. Microstate analysis is an alternative, and increasingly applied, EEG-representation based on topographic analysis. During EEG, the global electrical brain activity on the scalp remains semi-stable in successive short time periods, defined as EEG microstates, indicating the “quasi-simultaneity of activity among the nodes of large-scale networks” (7). These transient periods of stability last about 60 to 120 ms. The spatial distribution of the brain activity can be determined and plotted as EEG maps using multi-channel recording electrodes distributed across the scalp. Koenig et al. identified four clusters of map configurations and termed them classes A, B, C, and D. Microstate class A exhibits a left-right orientation, class B a right-left orientation, class C an anterior-posterior orientation, and class D a fronto-central maximum (see Figure 1) (8). These four cluster maps exhibited highly similar topographies in most subsequent studies, strongly resembling the maps initially described by Koenig et al. EEG microstates analysis were wildly used in neuropsychiatric diseases, such as schizophrenia (9, 10), dementia (10), narcolepsy (11), and stroke (12).

A resting-state functional MRI (rs-fMRI) study revealed that the brain can be intrinsically organized into large-scale functional networks in which the hemodynamic signature is stable for about 10 s (13). Spatial analyses of the topography of spontaneous EEGs also revealed similar discrete epochs of stable global brain states (so-called microstates), although those only remain quasi-stationary for about 100 ms (14, 15). Resting-state networks (RSNs) can be identified using both rs-fMRI and EEG, and several studies were conducted to investigate the association between the two modalities. The most direct way to investigate such associations is to use resting-state EEG and rs-fMRI simultaneously; such studies revealed that RSNs assessed using rs-fMRI were statistically significantly correlated with the temporal progression of all four EEG microstates (15). Two independent studies on combined EEG-fMRI appeared in the same issue of NeuroImage (15, 16). Britz et al. estimated the relationship between the EEG-defined microstates and the fMRI-defined resting states. The typical four EEG topographies determined by conventional EEG-microstate analysis dominated across all subjects. The convolution of the time course of these EEG topography maps with the hemodynamic response function allowed the authors to fit a linear model to the fMRI blood-oxygen-level-dependent (BOLD) responses, revealing four distinct distributed networks. The EEG networks were spatially correlated with the four RSNs identified using conventional fMRI ICA. Those RSNs were previously attributed to many aspects of cognitive processing such as “phonological processing, visual imagery, attention reorientation, and subjective interoceptive–autonomic processing” (15). Microstate A was related to negative BOLD activations of both superior and middle temporal parietal cortices, and regarded as the auditory or sensorimotor networks (15, 17). Microstate class B was related to negative BOLD activation in the striate, extrastriate, and both occipital cortices, and regarded as the visual network (15, 17). Microstate class C was mainly related to positive BOLD activation in both inferior frontal cortices, the dorsal anterior cingulate cortex, and the right insular area, and regarded as the salience network (15). Microstate class D exhibited correlations with negative BOLD activation in the right lateral ventral and dorsal regions of the frontal and parietal cortices, and regarded as an attentional network (15).

Scalp EEG is widely used in patients with epilepsy, most under task-free conditions. In most cases, EEG is only visually inspected for clinical use, and were analyzed by characterizing the temporal waveform morphology (18, 19) and/or frequency distribution of recordings at certain preselected electrodes (20), those methods may miss large parts of the information. In this study, we compared the differences in the resting-state EEG microstates in patients with IGE and healthy controls (HCs), to investigate the temporal dynamics of resting-state networks (RSNs) in patients with IGE and explore which information in resting-state EEGs may be clinically useful.

## Materials and Methods

### Participants

In the present study, patients were chosen from the clinic for epilepsy of the First Affiliated Hospital of Anhui Medical University in 2019. Diagnostic and syndromic classification were based on their clinical history and video-EEG findings, in accordance with the diagnostic criteria of the International League Against Epilepsy (1, 21). We included only patients with a normal cognitive ability (Montreal cognitive assessment [MoCA] score ≥26) and more than 5 years of education to ensure that they could understand the study procedures. Moreover, we included only patients with normal or corrected vision. Patients with a medical history of a psychiatric disorder and/or obvious cognitive disability were also excluded.

In total, 22 right-handed patients with IGE (only generalized tonic–clonic seizures alone) met the above criteria and were selected for this study. After detailed clinical and neurological examinations, all patients underwent continuous video-EEG recording for 24 h, as well as 3.0-Tesla high-resolution magnetic resonance imaging (MRI). Two of the patients were newly diagnosed with IGE and had not been treated with anti-seizure medicines (ASMs), while the other 20 patients were treated with ASMs. Nine patients were treated with valproic acid only, nine with lamotrigine only, and the remaining three with combination therapy. Of the 22 patients, 12 had been seizure-free for at least the previous two years (IGE-Seizure Free); the group with the remaining 10 patients was called “IGE-Seizure.” An additional HC group consisted of 17 right-handed, healthy participants who were matched to the patient group in terms of age, sex, and education.

All study procedures were approved by the Anhui Medical University Ethics Committee and conducted according to the Helsinki Declaration (1975 and subsequent revisions). Informed consent was obtained for all participants.

### Baseline Tests

All patients were asked to complete the MoCA, Beijing version. The Beck Depression Inventory (BDI, translated into Chinese) and Hamilton Anxiety Scale (HAMA, translated into Chinese) were also administered to obtain measurements of depressive and anxiety symptoms for all the participants.

### Resting-State EEG Recording and Preprocessing

Resting-state EEGs were recorded in a soundproof, light-controlled recording room, while participants were seated in a comfortable chair. All participants were instructed to keep their eyes closed and to relax during the whole recording period.

EEG signals were continuously recorded using the Curry 7 data acquisition system (Neuvo 64-channel Amplifier, Compumedics Neuroscan, Charlotte, NC, USA), with 64 scalp electrodes (Ag-AgCl) mounted on an elastic cap, in accordance with the extended International 10/20 system (22, 23), as well as two mastoid electrodes. An additional electrooculography (EOG) channel was used to monitor eye movements. All electrode impedances were maintained below 5 kΩ. The EEG and EOG activity values were continuously sampled at 1,000 Hz/channel and amplified with 0.01–100-Hz band-pass filtering. The EEG signals were monitored while being recorded. Participants were asked to briefly open their eyes if they appeared to become drowsy, after which the EEG recording was resumed. Each participant's EEG data was continuously recorded lasting for 10 to 20 min.

We used EEGLAB (Version 13.0.0b, http://sccn.ucsd.edu/eeglab/) to preprocess the EEG data; it is an open-source toolbox implemented in MATLAB (Version 2013b; The MathWorks, Inc., Natick, MA, USA). The continuous EEG data were downsampled to 500 Hz and segmented into 2-s epochs. Abnormal amplitude (≥100 μV) was defined as artifactual epochs and removed. Independent component analysis (ICA) was also used to correct artifacts such as eye movement, heartbeats, and muscle artifacts (60 ICA components were extracted and 0–2 ICA components were removed from each participant, removed components were decided by their spectrum and Spatio-temporal characteristics). Data were visually inspected to identify any epochs containing obvious eye-blink artifacts missed during the independent component analysis, and all epochs with interictal epileptiform discharges were rejected. Each participant's EEG data contain more than 30 epochs.

### Microstate Analysis

We performed microstate analysis according to the standard procedure described previously (24, 25). Before microstate analysis, the preprocessed EEG epochs were digitally band-pass-filtered between 1 and 40 Hz. For each EEG epoch, we calculated the global field power, which reflects the degree of variation in potential across electrodes at a time point. Subsequently, clustering analysis was applied to the local maximum of the global field power. Using a modified version of the K-mean clustering algorithm (26), four microstate class topographies were computed, namely microstate classes A, B, C, and D. This number of microstate classes was previously deemed optimal and was maintained in this study for compatibility with the existing literature (7). Three microstate parameters were computed, consisting of the average microstate duration (“Duration”), average number of microstates per-second (“Frequency”), and percentage of total analysis time occupied in that state (“Coverage”).

### Statistical Analysis

IBM SPSS Statistics for Windows, version 23.0 (IBM Corp., Armonk, NY, USA) was used for all statistical analyses. Non-parametric tests were performed to explore statistically significant differences in the background tests between the three groups, and sex differences between the three groups were assessed with chi-square tests.

Multivariate analyses of covariance (MANOVA) were performed for the three microstate parameters. Each MANOVA contained one between-subject factor for 3 groups (Seizure, Seizure free and HC groups), one within-subject factor for 4 microstate classes. When the main effects or interaction were significant, *Post hoc* ANOVA comparisons were considered. The threshold for significance was *p* < 0.05 for the MANOVA and the alpha value for Bonferroni procedures was 0.004 [0.05/ (3^*^4) = 0.004167].

## Results

### Demographic Information and Baseline Tests

Demographic data and the results of baseline tests of the three groups are summarized in Table 1. There were no statistically significant differences in age, education, MoCA, HAMA, or BDI between the three groups. The differences in disease duration between the two groups of patients were also not statistically significant. These details are provided in Table 1.

**Table 1:** Demographic data, background tests and executive functions between the three groups (Mean ± SD).

	**IGE-Seizure**	**IGE-Seizure Free**	**HC**	***P*** **-Value**
N	10	12	17	
Gender (male/female)	7/3	7/5	9/8	0.68
Age (years)	19.10 ± 5.34	20.00 ± 4.73	18.82 ± 1.29	0.41
Education (years)	10.80 ± 2.15	11.25 ± 3.25	12.41 ± 1.12	0.06
Disease duration (year)	3.23 ± 2.71	5.08 ± 1.98	–	0.07
Seizure frequency (events/year)	16.10 ± 18.11	–	–	
MoCA-B	27.60 ± 2.12	27.67 ± 1.78	28.59 ± 1.18	0.34
HAMA	1.70 ± 1.25	3.08 ± 3.32	2.06 ± 1.52	0.62
BDI	1.40 ± 1.84	2.50 ± 3.21	1.29 ± 1.10	0.69

### Microstate Characteristics

The four microstate classes accounted for 72.86% (standard deviation [SD]: 5.39%) of the global explained variance in the IGE-Seizure group, 75.43% (SD: 3.09%) in the IGE-Seizure Free group, and 74.11% (SD: 4.02%) in the HC group. Figure 1 shows the microstate topographic classes of the IGE-Seizure group, IGE-Seizure Free group and the HC group.

For Duration, MANOVA found that there was a significant interaction effect of 3 groups ^*^ 4 microstates (F =12.950, *p* < 0.001, Wilks' Lambda = 0.218, eta squared = 0.533), only microstate classes C showed significant difference between the three groups (F (2,36) = 9.753, *p* < 0.001); microstate classes A, B and D showed no significant difference (F (2,36) = 3.395, 3.494 and 2.266 respectively, *p* > 0.05*)*. Further analysis found that the duration of microstate class C in IGE-Seizure group were significantly shorter than in HC group (*p* < 0.001), but there were no significantly difference between HC and IGE-Seizure Free group, and between the IGE-Seizure and IGE-Seizure Free group (*p* = 0.429 and 0.025 respectively).

For Frequency, a significant interaction effect of 3 groups ^*^ 4 microstates were also found by MANOVA (F =10.365, *p* < 0.001, Wilks' Lambda = 0.273, eta squared = 0.478), only microstate classes A showed significant difference between the three groups (F (2,36) =8.385, *p* < 0.001); microstate classes B, C and D showed no significant difference (F (2,36) = 0.219, 3.925 and 0.975 respectively, *p* > 0.004*)*. Further analysis found that the frequency of microstate class A in IGE-Seizure group were significantly higher than in IGE-Seizure Free group (*p* < 0.001), but there was no significant difference between HC and IGE-Seizure Free group (*p* = 0.806), and between the HC and IGE-Seizure group (*p* = 0.009).

**Figure 2:**
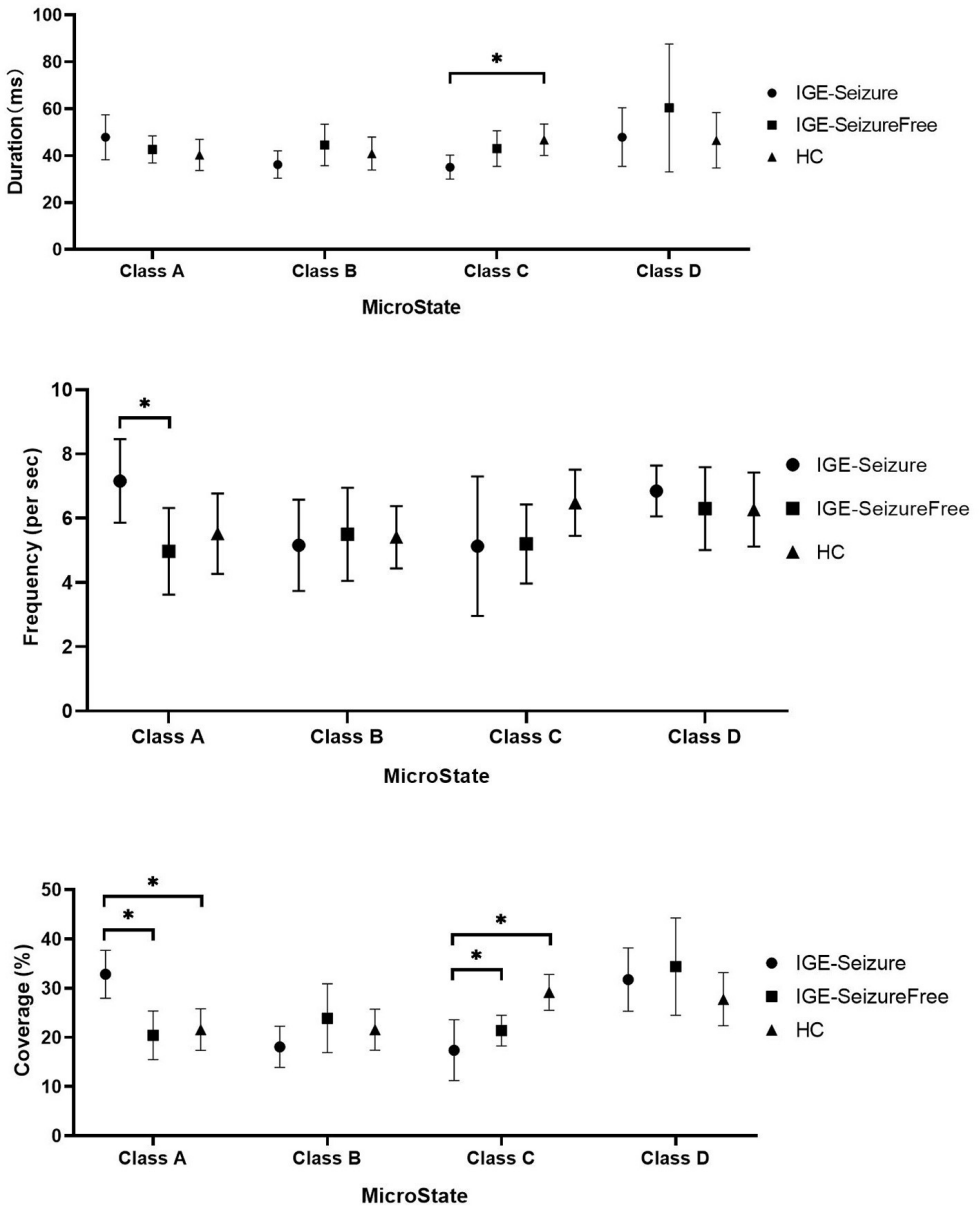
Microstate characteristics between the three groups. * Means *p* < 0.004 (*Post hoc* ANOVA).

For Coverage, which means the product of Duration and Frequency, MANOVA also found that there was a significant interaction effect of 3 groups ^*^ 4 microstates (F =13.638, *p* < 0.001, Wilks' Lambda = 0.206, eta squared = 0.546),microstate classes A and class C showed significant difference between the three groups (F (2,36) = 24.199, *p* < 0.001 and F (2,36) = 26.168, *p* < 0.001 respectively); The coverage of microstate class A in IGE-Seizure group were significantly higher than in HC group (*p* < 0.001) and in IGE-Seizure Free group (*p* < 0.001), but there were no significantly difference between HC and IGE-Seizure Free group; The coverage of microstate class C in IGE-Seizure group were significantly lower than in HC group (*p* < 0.001), and also the IGE-Seizure Free were significantly lower than HC group (*p* < 0.001), but there were no significantly difference between IGE-Seizure and IGE-Seizure Free group(*p* = 0.111); These details are provided in Table 2; Figure 2.

**Table 2:** Microstate characteristics between the three groups (Mean ± SD).

	**IGE-Seizure**	**IGE-Seizure** **Free**	**HC**	***P*****-Value** **(*****Post hoc*** **ANOVA)**
**Microstate classes A**				
Duration (ms)	47.86 ± 9.60	42.65 ± 5.76	40.33 ± 6.64	0.045
Frequency (per sec)	7.16 ± 1.30	4.97 ± 1.35	5.52 ± 1.25	0.001
Coverage (%)	32.81 ± 4.87	20.40 ± 4.93	21.57 ± 4.23	0.000
**Microstate classes B**				
Duration	36.21 ± 5.81	44.56 ± 8.82	40.91 ± 7.07	0.041
Frequency	5.16 ± 1.42	5.50 ± 1.45	5.41 ± 0.97	0.804
Coverage	18.07 ± 4.16	23.88 ± 6.97	21.56 ± 4.19	0.043
**Microstate classes C**				
Duration	35.10 ± 5.10	43.01 ± 7.57	46.76 ± 6.71	0.000
Frequency	5.13 ± 2.17	5.20 ± 1.23	6.48 ± 1.03	0.029
Coverage	17.38 ± 6.19	21.37 ± 3.12	29.14 ± 3.65	0.000
**Microstate classes D**				
Duration	47.91 ± 12.46	60.36 ± 27.25	46.54 ± 11.80	0.118
Frequency	6.85 ± 0.79	6.30 ± 1.29	6.27 ± 1.15	0.387
Coverage	31.73 ± 6.42	34.25 ± 9.89	27.73 ± 5.39	0.061

*The threshold for significance was p < 0.004 Post Hoc ANOVA*.

## Discussion

In the present study, we investigated the alterations of brain functional state dynamics in IGE patients and HCs using EEG microstate analysis to ascertain the temporal characteristics of sub-second brain activity. Four classes of microstates were identified in this study, according to those described in the existing literature. The main findings of this study can be summarized as follows. First, the mean Duration, Frequency, and Coverage of microstates B and D were similar between the three groups. Second, the Frequency and Coverage of microstate A were larger in patients with IGE with seizures in the previous 2 years than in those without such seizures and in HCs. Finally, the mean Duration and Coverage of microstate C were smaller in patients with IGE who had experienced seizures in the past 2 years than in those patients without seizures and in HCs. Those results may provide some insights into the aberrant intrinsic brain activities in IGE patients.

IGE patients presented no structural abnormalities on a standard brain magnetic resonance image, but they showed significant differences in the structural and functional connectivity when compared with healthy controls (27). The global efficiency, local efficiency, and clustering coefficients of the structural connectivity in patients with IGE were significantly decreased, whereas the characteristic path length and small-worldness index were increased (27). In patients with IGE, The global efficiency and local efficiency were increased and the small-worldness index decreased in the functional connectivity (27). Using graph theoretical network analysis derived from whole-brain magnetoencephalography recordings, patients with IGE showed a widespread increase in connectivity when compared to healthy controls (28). Because of the methodological differences, findings form previous studies should be interpreted with caution. Nevertheless, structural, and functional brain abnormalities may be present in IGE patients, results from current study that altered microstates in patients with IGE also support this idea.

Microstate class A was related to negative BOLD activations of both superior and middle temporal parietal cortices, and regarded as the auditory or sensorimotor networks (15, 17). Sensorimotor network was well-studied in epilepsy patients. Rolandic epilepsy is a form of well-characterized childhood epilepsy whose focal electroencephalographic abnormalities affect the same well-delineated local brain regions, rs-fMRI study found that the patients with Rolandic epilepsy exhibited less connectivity among the sensorimotor areas (29); another rs-fMRI study found that the synchrony was significantly higher in the sensorimotor network in IGE patients and in their unaffected relatives when compared to controls, also there was a trend toward higher synchrony in the generalized spike-wave discharges (GSW) network in patients and their unaffected relatives, those findings provide evidence that elevated fMRI BOLD synchrony in a sensorimotor network may be a state-independent endophenotype of IGE, present in patients in the absence of GSW, and present in unaffected relatives (30). In current study, the Frequency and Coverage of microstate A were larger in patients with IGE with seizures in the previous 2 years than in those without such seizures and in HCs, indicates that the sensorimotor network was altered in IGE patients and provide new evidence to support the previous rs-fMRI study (30).

Microstate class B was related to negative BOLD activation in the striate, extrastriate, and both occipital cortices, and regarded as the visual network (15, 17). Studies of visual network in patients with epilepsy were rare. Study of glioma-related epilepsy found that temporal lobe gliomas in the left hemisphere and glioma-related epilepsy altered visual networks in an opposing manner (31). In current study, we did not find any significant difference of microstate class B between the three groups, indicates that the visual network may be intact in patients with IGE.

Microstate class C was mainly related to positive BOLD activation in both inferior frontal cortices, the dorsal anterior cingulate cortex, and the right insular area, and regarded as the salience network (15). The salience network is a brain network comprised of the anterior insula and anterior cingulate cortex (32, 33). Compared with HCs, patients with focal epilepsy exhibited lower connectivity of the right fronto-insula cortex, insula, orbitofrontal cortex, inferior frontal cortex, and right medial prefrontal cortex, indicating that connectivity to the salience network was lower in patients with focal epilepsy (34). Patients with mesial temporal lobe epilepsy exhibited lower connectivity to both insulae and no connectivity to the dorsal anterior cingulate cortex (35). Patients with myoclonic seizures and those with absence seizures also exhibited lower functional connectivity of the salience network (36) than HCs. In the current study, the mean Duration and Coverage of microstate C were smaller in patients with IGE who experienced seizures in the past 2 years than in those that were seizure-free and in HCs. These results may mean that the salience network was altered in patients with IGE than in HCs, especially in those that had a recent history of seizures.

Microstate class D exhibited correlations with negative BOLD activation in the right lateral ventral and dorsal regions of the frontal and parietal cortices, and regarded as an attentional network (15). The attentional systems were proposed as two anatomically and functionally distinct systems in the human brain: a dorsal and a ventral frontoparietal system (37). Broadly speaking, the dorsal frontoparietal system was proposed to mediate the top-down, voluntary allocation of attention to locations or features, whereas the ventral frontoparietal system was proposed to be involved in detecting unattended or unexpected stimuli and triggering shifts of attention. The dorsal attention network (DAN) comprises the intraparietal sulcus and the frontal eye fields of each hemisphere and are supposedly organized bilaterally (38). The ventral attentional network comprises the temporoparietal junction and the ventral frontal cortex, typically responding when behaviorally relevant stimuli occur unexpectedly (38). Using rs-fMRI, Xiao et al. studied the attentional networks in patients with benign childhood epilepsy with centrotemporal spikes (BECTS) (39). They revealed that patients with BECTS and attention deficit hyperactivity disorder (ADHD) displayed lower functional connectivity in the DAN than patients with BECTS but without ADHD, and HCs. Patients with BECTS without ADHD exhibited higher functional connectivity in the DAN than HCs. Compared with HCs, both BECTS groups exhibited higher functional connectivity in the ventral attentional network. These results indicated that children newly diagnosed with BECTS displayed alterations in brain activity in the ventral and dorsal attentional networks (39). The DAN was also studied in patients with mesial temporal lobe epilepsy (40). Compared with HCs, patients with mesial temporal lobe epilepsy exhibited lower functional connectivity in almost all the regions of the DAN (40). In another study, network homogeneity in the right superior parietal lobule and right precuneus was statistically significantly higher in patients with right temporal lobe epilepsy than in HCs (41). Previous studies focused on focal epilepsy, in the current study, we did not find any significant difference between the IGE-seizure, IGE-seizure free and HC groups, suggesting that the attentional network may not be altered in patients with IGE, this is different from the existing research results. Inconsistent results of current study may indicate that RSNs identified by rs-fMRI cannot be equals to that identified by EEG.

The present study also has some limitations. The sample size is small and there may be some sampling bias. The influence of anti-epilepsy drugs was not considered. Also, we did not consider of the influence of focal seizure. In the future, more subjects will be included and various possible influencing factors will be fully considered.

## Conclusion

Using resting-state EEG microstate analysis, the present study indicated that the salience network and sensorimotor network were altered in patients with IGE (GTCS only), especially in those who had experienced seizures in the past 2 years.

## Data Availability Statement

The original contributions presented in the study are included in the article/supplementary material, further inquiries can be directed to the corresponding author/s.

## Ethics Statement

The studies involving human participants were reviewed and approved by Anhui Medical University Ethics Committee. Written informed consent to participate in this study was provided by the participants' legal guardian/next of kin.

## Author Contributions

YBJ and KW: designed experiments. MYZ and YH: carried out experiments. YBJ: analyzed experimental results, sequencing data, and wrote the manuscript. All authors contributed to the article and approved the submitted version.

## Funding

This study was supported by a grant from the University Natural Science Research Project of Anhui Province, China KJ2019A0289.

## Conflict of Interest

The authors declare that the research was conducted in the absence of any commercial or financial relationships that could be construed as a potential conflict of interest.

## Publisher's Note

All claims expressed in this article are solely those of the authors and do not necessarily represent those of their affiliated organizations, or those of the publisher, the editors and the reviewers. Any product that may be evaluated in this article, or claim that may be made by its manufacturer, is not guaranteed or endorsed by the publisher.

## References

[1] Scheffer IE Berkovic S Capovilla G Connolly MB French J Guilhoto L. ILAE classification of the epilepsies: position paper of the ILAE commission for classification and terminology. Epilepsia. (2017) 58:512–21. 10.1111/epi.13709 28276062 PMC5386840

[2] Nei M Bagla R. Seizure-related injury and death. Curr Neurol Neurosci Rep. (2007) 7:335–41. 10.1007/s11910-007-0051-1 17618541

[3] Li Q Cao W Liao X Chen Z Yang T Gong Q. Altered resting state functional network connectivity in children absence epilepsy. J Neurol Sci. (2015) 354:79–85. 10.1016/j.jns.2015.04.054 25982500

[4] Sharpee TO Destexhe A Kawato M Sekulić V Skinner FK Wójcik DK. 25th Annual computational neuroscience meeting: CNS-2016. BMC Neurosci. (2016) 17 Suppl 1:54. 10.1186/s12868-016-0283-6 27534393 PMC5001212

[5] Zhong C Liu R Luo C Jiang S Dong L Peng R. Altered structural and functional connectivity of juvenile myoclonic epilepsy: an fMRI study. Neural Plast. (2018) 2018:7392187. 10.1155/2018/7392187 29681927 PMC5846383

[6] Jia X Xie Y Dong D Pei H Jiang S Ma S. Reconfiguration of dynamic large-scale brain network functional connectivity in generalized tonic-clonic seizures. Hum Brain Mapp. (2020) 41:67–79. 10.1002/hbm.24787 31517428 PMC7267969

[7] Michel CM Koenig T. EEG microstates as a tool for studying the temporal dynamics of whole-brain neuronal networks: a review. Neuroimage. (2018) 180:577–93. 10.1016/j.neuroimage.2017.11.062 29196270

[8] Koenig T Lehmann D Merlo MC Kochi K Hell D Koukkou M. deviant EEG brain microstate in acute, neuroleptic-naive schizophrenics at rest. Eur Arch Psychiatry Clin Neurosci. (1999) 249:205–11. 10.1007/s004060050088 10449596

[9] Irisawa S Isotani T Yagyu T Morita S Nishida K Yamada K. Increased omega complexity and decreased microstate duration in nonmedicated schizophrenic patients. Neuropsychobiology. (2006) 54:134–9. 10.1159/000098264 17199099

[10] Nishida K Morishima Y Yoshimura M Isotani T Irisawa S Jann K. EEG microstates associated with salience and frontoparietal networks in frontotemporal dementia, schizophrenia and Alzheimer's disease. Clin Neurophysiol. (2013) 124:1106–14. 10.1016/j.clinph.2013.01.005 23403263

[11] Drissi NM Szakacs A Witt ST Wretman A Ulander M Stahlbrandt H. Altered brain microstate dynamics in adolescents with narcolepsy. Front Hum Neurosci. (2016) 10:369. 10.3389/fnhum.2016.00369 27536225 PMC4971065

[12] Zappasodi F Croce P Giordani A Assenza G Giannantoni NM Profice P. Prognostic value of EEG microstates in acute stroke. Brain Topogr. (2017) 30:698–710. 10.1007/s10548-017-0572-0 28547185

[13] Fox MD Snyder AZ Vincent JL Corbetta M Van Essen DC Raichle ME. The human brain is intrinsically organized into dynamic, anticorrelated functional networks. Proc Natl Acad Sci U S A. (2005) 102:9673–8. 10.1073/pnas.0504136102 15976020 PMC1157105

[14] Lehmann D Ozaki H Pal I. EEG alpha map series: brain micro-states by space-oriented adaptive segmentation. Electroencephalogr Clin Neurophysiol. (1987) 67:271–88. 10.1016/0013-4694(87)90025-3 2441961

[15] Britz J Van De Ville D Michel CM. BOLD correlates of EEG topography reveal rapid resting-state network dynamics. Neuroimage. (2010) 52:1162–70. 10.1016/j.neuroimage.2010.02.052 20188188

[16] Musso F Brinkmeyer J Mobascher A Warbrick T Winterer G. Spontaneous brain activity and EEG microstates. A novel EEG/fMRI analysis approach to explore resting-state networks. Neuroimage. (2010) 52:1149–61. 10.1016/j.neuroimage.2010.01.093 20139014

[17] Yuan H Zotev V Phillips R Drevets WC Bodurka J. Spatiotemporal dynamics of the brain at rest–exploring EEG microstates as electrophysiological signatures of BOLD resting state networks. Neuroimage. (2012) 60:2062–72. 10.1016/j.neuroimage.2012.02.031 22381593

[18] van Graan LA Lemieux L Chaudhary UJ. Methods and utility of EEG-fMRI in epilepsy. Quant Imaging Med Surg. (2015) 5:300–12. 25853087 10.3978/j.issn.2223-4292.2015.02.04 PMC4379314

[19] Jayalakshmi S Dhondji M Vooturi S Patil A Vadapalli R. Inter-ictal EEG patterns in malformations of cortical development and epilepsy. Clin Neurol Neurosurg. (2020) 196:106022. 10.1016/j.clineuro.2020.106022 32599425

[20] Jiang Y Zhu M Yu F Wang K. Impaired empathy in patients with idiopathic generalized epilepsy: an event-related potentials study. Epilepsy Behav. (2020) 111:107274. 10.1016/j.yebeh.2020.107274 32693373

[21] Fisher RS Acevedo C Arzimanoglou A Bogacz A Cross JH Elger CE. ILAE official report: a practical clinical definition of epilepsy. Epilepsia. (2014) 55:475–82. 10.1111/epi.12550 24730690

[22] Nuwer MR Comi G Emerson R Fuglsang-Frederiksen A Guerit JM Hinrichs H. IFCN standards for digital recording of clinical EEG the international federation of clinical neurophysiology. Electroencephalogr Clin Neurophysiol Suppl. (1999) 52:11–4. 10590972

[23] Nuwer MR Comi G Emerson R Fuglsang-Frederiksen A Guerit JM Hinrichs H. IFCN standards for digital recording of clinical EEG. International federation of clinical neurophysiology. Electroencephalogr Clin Neurophysiol. (1998) 106:259–61. 10.1016/S0013-4694(97)00106-5 9743285

[24] Cai Y Chen S Chen Y Li J Wang CD Zhao F. Altered Resting-State EEG Microstate in Idiopathic Sudden Sensorineural Hearing Loss Patients With Tinnitus. Front Neurosci. (2019) 13:443. 10.3389/fnins.2019.00443 31133786 PMC6514099

[25] Kikuchi M Koenig T Munesue T Hanaoka A Strik W Dierks T. EEG microstate analysis in drug-naive patients with panic disorder. PLoS ONE. (2011) 6:e22912. 10.1371/journal.pone.0022912 21829554 PMC3146502

[26] Pascual-Marqui RD Michel CM Lehmann D. Segmentation of brain electrical activity into microstates: model estimation and validation. IEEE Trans Biomed Eng. (1995) 42:658–65. 10.1109/10.391164 7622149

[27] Lee DA Kim BJ Lee HJ Kim SE Park KM. Network characteristics of genetic generalized epilepsy: are the syndromes distinct? Seizure. (2020) 82:91–8. 10.1016/j.seizure.2020.09.022 33045541

[28] Elshahabi A Klamer S Sahib AK Lerche H Braun C Focke NK. Magnetoencephalography reveals a widespread increase in network connectivity in idiopathic/genetic generalized epilepsy. PLoS ONE. (2015) 10:e0138119. 10.1371/journal.pone.0138119 26368933 PMC4569354

[29] Xiao F Lei D An D Li L Chen S Chen F. Functional brain connectome and sensorimotor networks in rolandic epilepsy. Epilepsy Res. (2015) 113:113–25. 10.1016/j.eplepsyres.2015.03.015 25986198

[30] Tangwiriyasakul C Perani S Abela E Carmichael DW Richardson MP. Sensorimotor network hypersynchrony as an endophenotype in families with genetic generalized epilepsy: A resting-state functional magnetic resonance imaging study. Epilepsia. (2019) 60:e14–9. 10.1111/epi.14663 30730052 PMC6446943

[31] Fang S Zhou C Fan X Jiang T Wang Y. Epilepsy-Related Brain Network Alterations in Patients With Temporal Lobe Glioma in the Left Hemisphere. Front Neurol. (2020) 11:684. 10.3389/fneur.2020.00684 32765403 PMC7380082

[32] Seeley WW Menon V Schatzberg AF Keller J Glover GH Kenna H. Dissociable intrinsic connectivity networks for salience processing and executive control. J Neurosci. (2007) 27:2349–56. 10.1523/JNEUROSCI.5587-06.2007 17329432 PMC2680293

[33] Menon V Uddin LQ. Saliency, switching, attention and control: a network model of insula function. Brain Struct Funct. (2010) 214:655–67. 10.1007/s00429-010-0262-0 20512370 PMC2899886

[34] Shu T Xiao X Long Z Zhang R. Reduced structural covariance connectivity of defaut mode network and salience network in MRI-normal focal epilepsy. Neuroreport. (2020) 31:1289–95. 10.1097/WNR.0000000000001541 33165193

[35] Burianova H Faizo NL Gray M Hocking J Galloway G Reutens D. Altered functional connectivity in mesial temporal lobe epilepsy. Epilepsy Res. (2017) 137:45–52. 10.1016/j.eplepsyres.2017.09.001 28923408

[36] Li Q Chen Y Wei Y Chen S Ma L He Z. Functional Network Connectivity Patterns between Idiopathic Generalized Epilepsy with Myoclonic and Absence Seizures. Front Comput Neurosci. (2017) 11:38. 10.3389/fncom.2017.00038 28588471 PMC5440462

[37] Corbetta M Shulman GL. Control of goal-directed and stimulus-driven attention in the brain. Nat Rev Neurosci. (2002) 3:201–15. 10.1038/nrn755 11994752

[38] Vossel S Geng JJ Fink GR. Dorsal and ventral attention systems: distinct neural circuits but collaborative roles. Neuroscientist. (2014) 20:150–9. 10.1177/1073858413494269 23835449 PMC4107817

[39] Xiao F Li L An D Lei D Tang Y Yang T. Altered attention networks in benign childhood epilepsy with centrotemporal spikes (BECTS): A resting-state fMRI study. Epilepsy Behav. (2015) 45:234–41. 10.1016/j.yebeh.2015.01.016 25825370

[40] Zhang Z Lu G Zhong Y Tan Q Yang Z Liao W. Impaired attention network in temporal lobe epilepsy: a resting FMRI study. Neurosci Lett. (2009) 458:97–101. 10.1016/j.neulet.2009.04.040 19393717

[41] Zhou S Xiong P Ren H Tan W Yan Y Gao Y. Aberrant dorsal attention network homogeneity in patients with right temporal lobe epilepsy. Epilepsy Behav. (2020) 111:107278. 10.1016/j.yebeh.2020.107278 32693375

